# The Unusual Suspect: A Case of Non-occlusive Mesenteric Ischemia in a Patient With Cirrhosis

**DOI:** 10.4021/gr226w

**Published:** 2010-09-20

**Authors:** Muhammad Z. Bawany, Ali Nawras, Wael I. Youssef, Thomas Sodeman

**Affiliations:** a Division of Internal Medicine, University of Toledo Medical Center, Toledo, Ohio, USA; b Division of Gastroenterology and Hepatology, University of Toledo Medical Center, Toledo, Ohio, USA

**Keywords:** Non-occulusive mesenteric ischemia, Cirrhosis, Jejunal infarcation

## Abstract

Acute mesenteric ischemia has a variety of etiologies. Non-occulusive mesenteric ischemia accounts for 20-30% of patients with acute mesenteric ischemia. We describe a case of non-occulusive jejunal ischemia leading to infarction that occurred in a patient with cirrhosis and no previous history of cardiovascular disease.

## Introduction

Acute mesenteric ischemia (AMI) has a variety of etiologies. Non-occulusive mesenteric ischemia (NOMI) accounts for 20-30% of patients with AMI [1]. We describe a case of non-occulusive mesenteric ischemia leading to infarction that occurred in a patient with cirrhosis.

## Case Report

A 53 years old female was brought to emergency department with abdominal pain and altered mental status (AMS). Her medical history was significant for chronic alcoholism, tobacco abuse and hepatitis C. Her blood pressure upon presentation was 101/64 mmHg, pulse 109 beats/min, respirations 22breaths/min, oxygen saturation of 97% on 4 liters oxygen per minute and she was afebrile. On examination she had generalized yellow discoloration of the body. Abdominal examination was significant for right upper quadrant tenderness, hepatosplenomegaly and absence of bowel sounds. The rest of the examination was unremarkable.

Her initial laboratory showed Hb 14.5 g/dl, WBC 10.2 thousand/mm^3^, platelets 106,000 thousand/mm^3^, Na 116 meq/L, K 2.9 meq/L, BUN 13 mg/dl, Cr 2.7 mg/dl, CK-MB 18 ng/ml and troponin 0.08 ng/ml, ammonia 168 umol/L, bilirubin 29.2 mg/dl with direct bilirubin of 18.5 mg/dl, albumin 2.8 g/dl, INR 2.34, alkaline phosphatase 270 IU/L, ALT 172 IU/L, AST 679 IU/L and lactate of 10.5 mmol/L. Abdominal ultrasound showed an enlarged liver with distended gall bladder. She was admitted to Intensive Care Unit (ICU) because of AMS. The patient started having coffee ground emesis within an hour of admission to the ICU. A nasogastric tube was placed which recovered 600 ml of coffee ground aspirate. She gradually became hypotensive and was subsequently started on pressors.

**Figure 1:**
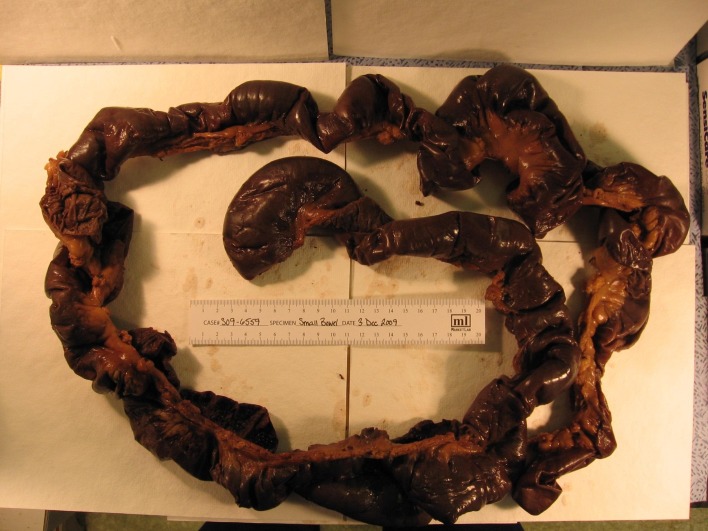
Gross specimen of resected small intestine.

The patient underwent an emergent esophagogastroduodenoscopy which revealed severe reflux esophagitis without active bleeding in the distal esophagus and no varices. The duodenum was erythematous and eccymotic, consistent with ischemia. General surgery was consulted and patient underwent an exploratory laparotomy which revealed dark brown serosa of jejunum 3 cm distal to the ligament of Treitz extending distally to approximately 180 cm sparing the ileum (Fig. 1). The infracted bowel was resected. Liver was found to be massively enlarged with multiple small nodules grossly suspected of cirrhosis and a needle biopsy was done. The colon was normal on visual inspection. Her abdomen was left open as her intestine was in discontinuity as well as for a possible second look in 24 - 48 hours.

Postoperatively the patient was continued on multiple pressors as she was still profoundly hypotensive. As her condition continued to decline and showed no signs of improvement on mechanical ventilation, support was withdrawn according to her wishes.

**Figure 2:**
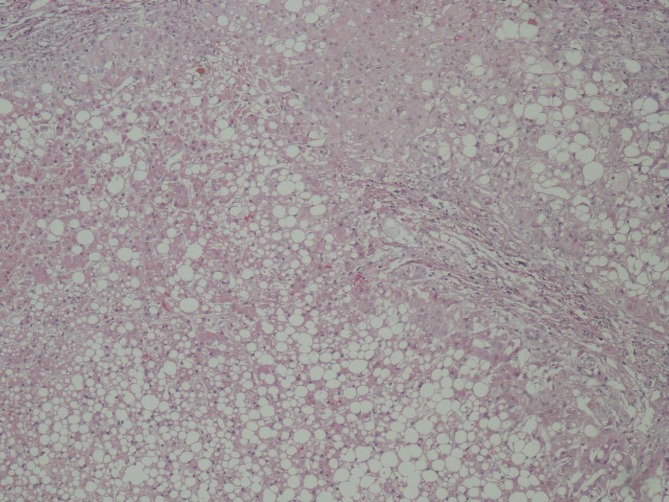
Liver biopsy showing macrosteatosis.

Pathology of the specimen showed an extensive full thickness ischemic necrosis of the intestine without any significant evidence of atherosclerosis or thrombus in the vessels, consistent with NOMI. The liver biopsy confirmed cirrhosis with macrovesicular steatosis and necrosis (Fig. 2).

## Discussion

To our knowledge, this is the first case report of NOMI in a patient with cirrhosis. We postulate this to be secondary to elevated levels of neurohormonal mediators like vasopressin and angiotensin, as a result of abnormal metabolism, causing hypoperfusion and vasoconstriction [2].

NOMI commonly occurs in an elderly person with cardiovascular disease (CVD) on medical therapy, as opposed to our patient who was not on any medications for CVD. Cases have been reported with cocaine use [3, 4]; however, our patient’s urine toxicology was negative. Mortality rate with NOMI is nearly 70%, usually because of a delay in diagnosis and treatment for the causes of ischemia [5]. Small intestinal villous tips are most vulnerable to the ischemia and after 3 - 6 hours [6], this mucosal damage can lead to translocation of bacteria and sepsis [7].

This case is of particular interest because neither our patient had any history of CVD, which has been thought to be one of the risk factor for NOMI, nor she had a thrombus or significant atherosclerosis in her mesenteric vessel, which has been reported in the past to be the cause of mesenteric ischemia in a patient with cirrhosis. However, more work needs to be done to better understand the pathophysiology of NOMI in a patient with cirrhosis.
